# A Video-Based Artificial Intelligence Platform for Measuring Esophagogastroduodenoscopy Duration and Suggesting Real-Time Landmark Documentation

**DOI:** 10.1055/a-2965-6499

**Published:** 2026-10-13

**Authors:** Zohar Levi, Shani Brosh, Manor Shpriz, Ziv Varsano, Daniel Khodyrker, Dotan Asselman, Sari Maril, Keren Hod, Hadar Edelman-Klapper, Sapir Eizenstein, Ian M. Gralnek, Inbar Segol, Naim Abu-Freha

**Affiliations:** 1 Gastroenterology 36632 Rabin Medical Center Beilinson HospitalPetah TikvaCenter DistrictIsrael; 2 Gray Faculty of Medical and Health Sciences 26745 Tel Aviv UniversityTel Aviv-YafoTel Aviv DistrictIsrael; 3 Division of Medicine 64850 Assuta Medical CenterTel Aviv-YafoTel Aviv DistrictIsrael; 4 Medicine 58880 Technion Israel Institute of Technology The Ruth and Bruce Rappaport Faculty of MedicineHaifaHaifa DistrictIsrael; 5 Data Based Clinical Quality Unit 64850 Assuta Medical CenterTel Aviv-YafoTel Aviv DistrictIsrael; 6 Unit of Big Data Science 64850 Assuta Medical CenterTel Aviv-YafoTel Aviv DistrictIsrael; 7 Research and DevelopmentTheator, Inc.Palo AltoCaliforniaUnited States; 8 Department of Nutrition Sciences 42732 Ariel UniversityArielIsrael; 9 Department of Academy and Research 64850 Assuta Medical CenterTel Aviv-YafoTel Aviv DistrictIsrael; 10 Gray Faculty of Medical and Health SciencesTel Aviv UniversityTel Aviv-YafoTel Aviv DistrictIsrael; 11 Epidemiology 58408 Tel Aviv University Sackler Faculty of MedicineTel AvivTel AvivIsrael; 12 Gastroenterology 61172 Ha'emek HospitalAfulaNorth DistrictIsrael; 13 Medicine 26732 Ben-Gurion University of the NegevBe'er ShevaSouth DistrictIsrael; 14 Gastroenterology InstituteAssuta Medical CenterBeer-ShevaIsrael

**Keywords:** quality and logistical aspects, quality management, image and data processing, documentation, endoscopy upper GI tract

## Abstract

**Background and Study Aims** The European Society of Gastrointestinal Endoscopy (ESGE) sets key quality indicators for esophagogastroduodenoscopy (EGD), including a minimum procedural time of 7 min and photodocumentation of landmarks. We evaluated AI-derived procedural times, physician variations, and the impact of AI suggestions on landmark documentation rates.

**Methods** This retrospective multicenter study included 78,336 EGDs performed by 213 physicians (01.01.2024–31.12.2025). A video-based AI platform (Theator Inc., Palo Alto, USA) measured procedural time, provided real-time landmark suggestions, and measured landmark documentation. Two blinded experts performed confirmatory validation of 500 procedures, with concordance defined as agreement within 2 s.

**Results** Median procedural time was 3.30 min (IQR: 2.27–4.65). AI timestamps were concordant with expert review in 98.5% of cases (95% CI: 97.1–99.3%). Among 203 physicians performing at least 50 procedures, procedural time varied from 1.35 to 7.56 min (a 5.6-fold difference), with only 6.1% of procedures meeting the ESGE-recommended 7-min threshold. AI landmark suggestions reduced zero-landmark procedures by 42.6% (15.8 vs. 27.5%; *P* < 0.001), with improvements across all seven anatomical sites ( *P* < 0.001 for each). Physicians in the slowest total EGD time quintile had fewer normal examinations than those in the fastest quintile: 19.8 versus 31.7% ( *P* = 0.002), an association that persisted when physicians were restratified by biopsy-free procedural time.

**Conclusions** Video-based AI accurately measured EGD procedure times and improved landmark documentation rate with AI-generated suggestions. A median procedural time of 3.3 min reveals a significant gap in quality standards. Automated, unattended measurement offers a scalable route to auditing upper endoscopy quality.

## Introduction

High-quality esophagogastroduodenoscopy (EGD) requires careful mucosal inspection and systematic photodocumentation of key anatomical landmarks. 1 2 3 4 These process measures are increasingly recognized as core determinants of procedural rigor because upper gastrointestinal neoplasia can be subtle and easily missed, and its detection is influenced by the thoroughness of the examination. 1 2 3 4 Recent guidelines, consensus statements, and expert reviews emphasize adequate inspection time, complete visualization of relevant anatomical sites, and structured photodocumentation as essential elements of high-quality upper endoscopy. 1 2 3 4 Guidelines recently published by the European Society of Gastrointestinal Endoscopy (ESGE) define 7 min as the procedural duration required for adequate inspection. 3

Still, the quality of upper endoscopy remains difficult to measure in routine clinical practice. Manual capture of procedural duration is labor-intensive, and the completeness of photodocumentation is often recorded inconsistently and is difficult to audit at scale. 1 2 Clinical studies show that longer examination time is associated with improved detection of focal lesions and upper gastrointestinal neoplasia, supporting procedural time as a meaningful quality indicator. 5 6 7 8 At the same time, upper endoscopy quality remains operator dependent, highlighting the need for scalable systems that can measure quality more objectively and consistently across physicians and sites. 2 3

Artificial intelligence (AI) offers a practical way to address these challenges. Early studies of AI-assisted systems demonstrated that computer vision can improve the quality of upper endoscopy by reducing blind spots and supporting systematic examination during EGD. 9 10 Subsequent work expanded these capabilities to include automated image reporting, photodocumentation support, and AI-assisted quality monitoring. 11 12 13 14 Together, findings suggest that AI may serve not only as a detection aid, but also as a tool for procedural quality measurement and standardization in routine clinical practice. 11 12 13 14 15

However, critical gaps remain. Much of the published literature has focused on technical development, prospective assistance systems, or controlled trial settings. 9 10 11 12 13 14 15 Meanwhile, evidence on large-scale automated auditing of procedural time and photodocumentation in routine, multicenter community practice remains limited. In addition, the effect of AI-generated landmark suggestions on real-world documentation behavior has not been well characterized at an operational scale. In the current study, we examined a video-based AI platform that automates procedural time measurement and provides real-time landmark suggestions, using it to assess physician-level variation in practice and its potential to improve photodocumentation rates.

## Methods

### Study Design and Setting

Assuta Medical Centers is the largest provider of endoscopic procedures in Israel and functions as the procedural arm of Maccabi Healthcare Services, one of Israel’s national health funds. The great majority of procedures in this cohort were performed on patients insured under the national health system, who were evaluated by gastroenterologists in community clinics and referred for endoscopy at an Assuta unit under public reimbursement. Only a minority of procedures are driven by private or supplementary insurance. Payer status was not recorded at the individual procedure level and could not be analyzed as a covariate. In this retrospective observational study, we analyzed EGD procedures performed across 23 endoscopy units between January 2024 and December 2025.

All 23 endoscopy units are nonacademic service units. None is a university teaching unit, no trainees participated in the procedures analyzed, and all examinations were performed by board-certified gastroenterologists.

The cohort included 213 endoscopists who performed diagnostic and screening EGD. All procedures used the Theator AI platform (Theator Inc., Palo Alto, California, USA) for real-time documentation assistance and automated capture of quality metrics. Procedures with a duration under 30 s were excluded. The Assuta Medical Centers institutional review board approved the study, waiving the requirement for informed patient consent (approval no.: 0002-23 ASMC).

### AI Platform and Automated Measurements

The Theator AI platform is a computer vision-based surgical intelligence system integrated directly with the endoscopic camera tower, capturing and processing endoscopic video in real time. The platform employs deep learning models that process endoscopic video as a continuous sequence of frames, using a Video Transformer Network (VTN) to extract features. The VTN uses attention-based modules that capture both spatial and temporal information across the video stream. These features are subsequently passed to a Long Short-Term Memory (LSTM) network to detect discrete visual events. Models were developed and validated on large manually annotated datasets of minimally invasive surgical video across multiple procedure types, with performance assessed against human annotations as the reference standard on independent test sets. For procedural time measurement, this architecture automatically identifies in-body intubation and scope extubation events from the video feed, calculating procedural time with millisecond precision without manual input. For landmark documentation, the same framework continuously analyzes the video and detects predefined anatomical structures in real time. When a landmark is detected, the platform presents a real-time documentation suggestion on the procedure room display, allowing the endoscopist to confirm or dismiss the suggestion with a single click. The platform specifically tracks seven key anatomical landmarks: esophagus, gastroesophageal junction, gastric cardia/fundus, corpus/body, angulus/incisura, pylorus/antrum, and second portion of duodenum. The system operates passively and does not interfere with the endoscopist’s workflow or clinical decision-making.

### Platform Validation and Data Collection

The algorithm underlying the automated timestamps detects the moment the endoscope enters and exits the patient’s body: esophageal intubation and extubation in the context of EGD. This algorithm has been previously published and validated in a peer-reviewed study 16 and has demonstrated near-perfect accuracy in commercial deployment across multiple surgical specialties and procedure types. To validate platform-based procedural times in the EGD setting, we sequentially selected 500 EGDs to undergo confirmatory review by two independent board-certified gastroenterologists blinded to AI-derived timestamps (Z.L., N.A.-F.). This review was intended as a confirmatory assessment of algorithm equivalence in the EGD setting, rather than a de novo validation of an unproven method. Reviewers manually reviewed videos frame by frame and annotated intubation and extubation times. Agreement was defined as concordance within a 2-s margin. Each procedure was independently assessed by both reviewers. Photodocumentation completeness was verified by comparing AI-identified landmarks with the images stored in the endoscopy reporting system, confirming that the platform record corresponded to what was actually saved. This was a concordance check of presence or absence only; no assessment of image quality or diagnostic adequacy was performed.

For each procedure, we extracted the physician identifier, patient age and gender, procedural time, number of anatomical landmarks documented, number of biopsies acquired, and endoscopic diagnoses. The AI platform was active for all 78,336 included procedures, providing automated procedural time measurement throughout. The landmark documentation suggestion feature was deployed in a phased rollout over the study period. AI-generated landmark suggestions were therefore available in 46,911 procedures (59.9%) and not available in the remaining 31,425 procedures (40.1%), creating two naturally occurring comparison groups within the same AI platform deployment. The comparison of the photodocumentation rate between these groups reflects the incremental effect of the active landmark suggestion feature.

### Statistical Analysis

Descriptive statistics included the median and interquartile range (IQR) for continuous variables and frequencies and percentages for categorical variables. For between-group comparisons, we used the Mann–Whitney *U* test, chi-square test, and Kruskal–Wallis test as appropriate. We stratified procedural times into quintiles at both the procedure level and the physician level, and categorized patient age (0–17, 18–29, 30–39, 40–49, 50–59, 60–69, 70–79, 80 years and above) and biopsy count (0, 1–2, 3–5, >5 biopsies). Spearman’s rank correlation was used for associations between continuous variables. Physician-level practice variation was quantified using the coefficient of variation (CV). To assess the association between AI-derived procedural time and endoscopic diagnostic yield, physicians were stratified into quintiles based on their median total EGD time across all procedures. Total procedural time includes time spent evaluating, photographing, and sampling abnormalities. An association between duration and documented findings may therefore reflect the presence of the finding rather than the thoroughness of inspection. In a subanalysis, physicians were restratified into quintiles of biopsy-free procedural time, defined as each physician’s median duration across procedures in which no biopsy was taken, so that the exposure could not be inflated by sampling activity. Physicians with at least 30 such procedures were included, and the proportion of examinations coded as normal was compared across quintiles using all of that physician’s procedures with available diagnosis data, including those in which a biopsy was taken. Trend across quintiles was tested with the Jonckheere–Terpstra test on physician-level proportions, and the association with biopsy-free procedural time as a continuous variable with Spearman correlation. All tests were two-tailed with significance set at *P* < 0.05. Analyses were performed using IBM SPSS Statistics for Windows, version 29 (IBM Corp., Armonk, NY, USA).

## Results

### Study Population and Platform Validation

After excluding 140 EGDs with procedural duration under 30 s, we analyzed 78,336 EGDs performed by 213 physicians across 23 endoscopy rooms. Median patient age was 55.1 years (IQR: 41.3–66.4), and 65.4% of procedures were performed in patients aged 45 years or older. The intubation and extubation detection algorithm has been previously validated and published. 16 In the current confirmatory assessment, among the 500 validation procedures, AI-derived timestamps showed 98.5% concordance with expert manual review (Physician A: 492/500 [98.4%, 95% CI: 97.1–99.2%]; Physician B: 493/500 [98.6%, 95% CI: 97.3–99.3%]). A single discordant case between the two reviewers was classified as nonconcordant; no systematic timing errors were identified. AI-based landmark identification corresponded to the saved photodocumentation in all reviewed cases. This confirms concordance of the documentation record and does not constitute an assessment of image quality.

### Procedural-Time Distribution and Physician-Level Variation

Overall median procedural time was 3.30 min (IQR: 2.27–4.65). At the procedure level, median times across quintiles were 1.65 min in Q1, 2.48 min in Q2, 3.32 min in Q3, 4.35 min in Q4, and 6.25 min in Q5 ( Fig. 1A ; Table 1 ).

**Fig. 1:**
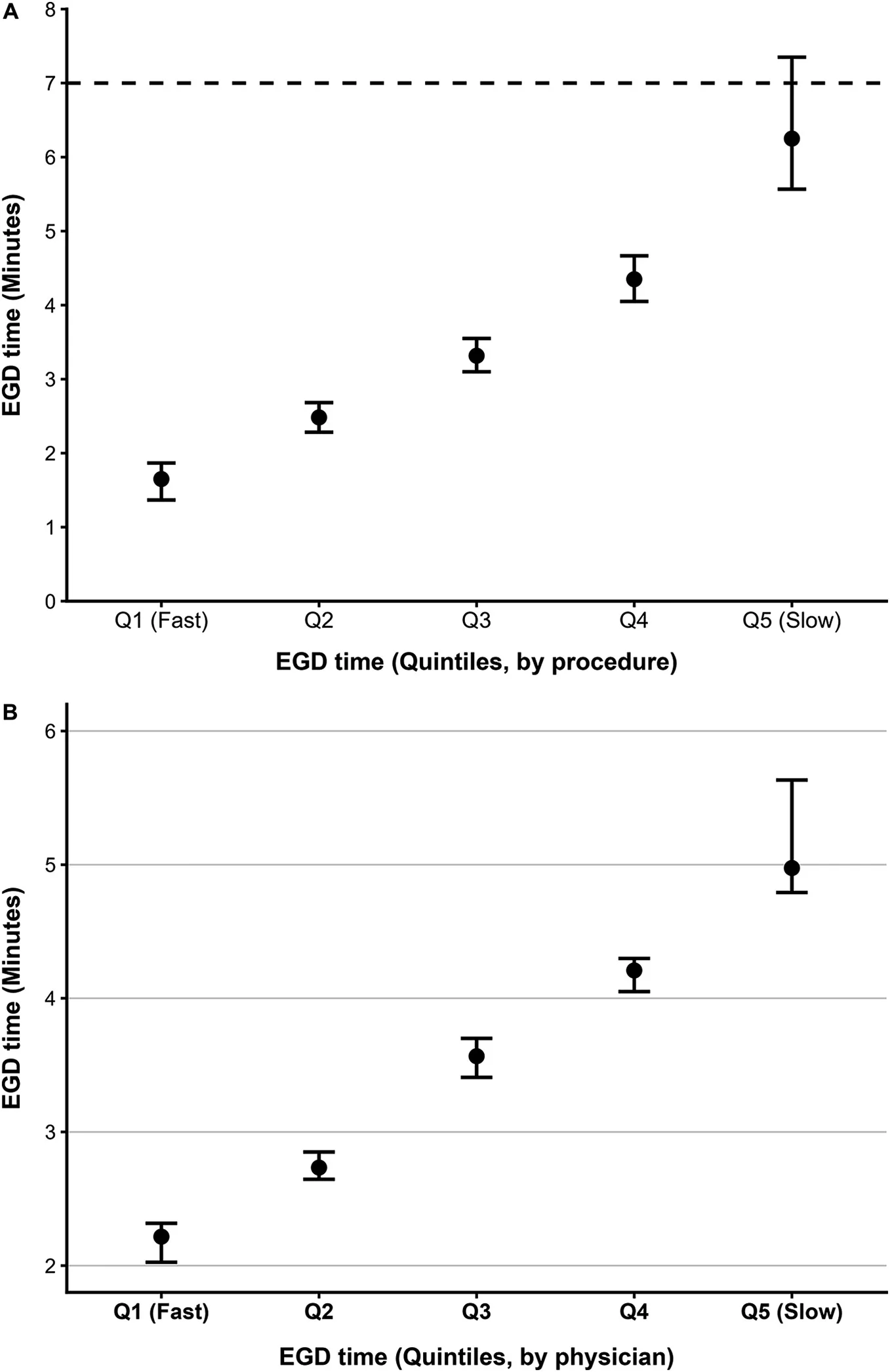
Procedural-time distribution across the study cohort. ( **A** ) Box plots of EGD procedural time across procedure-level quintiles (Q1–Q5; *n* = 78,336). Median times were 1.65, 2.48, 3.32, 4.35, and 6.25 min in Q1–Q5, respectively. The dashed horizontal line indicates the ESGE-recommended 7-min threshold. ( **B** ) Distribution of physician-level median procedural times across physician quintiles ( *n* = 203 physicians with ≥50 procedures). Quintile medians were 2.22, 2.73, 3.57, 4.21, and 4.97 min in Q1–Q5, respectively. EGD, esophagogastroduodenoscopy; ESGE, European Society of Gastrointestinal Endoscopy; IQR, interquartile range.

**Table 1:** Distribution of procedural time across procedure-level quintiles ( *n* = 78,336).

Quintile	*N*	%	Median (min)	IQR (min)	Mean landmarks
Q1 (Fastest)	15,902	20.3	1.65	1.37–1.87	1.78
Q2	15,604	19.9	2.48	2.28–2.68	2.27
Q3	15,596	19.9	3.32	3.10–3.55	2.52
Q4	15,936	20.3	4.35	4.05–4.67	2.62
Q5 (Slowest)	15,298	19.5	6.25	5.57–7.35	2.72
Total	78,336	100.0	3.30	2.27–4.65	2.38

IQR, interquartile range.

Among the 203 physicians who performed at least 50 procedures, median procedural times ranged from 1.35 to 7.56 min, representing a 5.6-fold variation (CV 33.3%). When physicians were stratified into quintiles by their median procedural time, quintile medians were 2.22, 2.73, 3.57, 4.21, and 4.97 min in Q1–Q5, respectively ( Fig. 1B ; Table 2 ).

**Table 2:** Distribution of physician median procedural time across physician-level quintiles ( *n* = 203).

Quintile	Physicians *N* (%)	Procedures *N* (%)	Median of medians (min)	IQR (min)
Q1 (Fastest)	41 (20.2)	19,297 (24.7)	2.22	2.02–2.32
Q2	40 (19.7)	15,459 (19.8)	2.73	2.65–2.85
Q3	41 (20.2)	17,058 (21.8)	3.57	3.41–3.70
Q4	40 (19.7)	13,023 (16.7)	4.21	4.05–4.30
Q5 (Slowest)	41 (20.2)	13,277 (17.0)	4.97	4.79–5.63
Total	203 (100.0)	78,114 (100.0)	3.57	2.64–4.31

IQR, interquartile range. Only physicians with at least 50 procedures were included in physician-level analyses.

Recent ESGE quality guidelines define 7 min as the recommended minimum procedural duration for adequate EGD inspection. 3 Against this benchmark, only 4,770 procedures in the current cohort (6.1%) lasted 7 min or more. Procedures lasting at least 5 min accounted for 20.8% of the cohort, and those lasting at least 6 min for 11.4%. The 7-min threshold was met exclusively within the slowest procedure-level quintile (Q5; 31.2%), with no procedures in quintiles Q1 through Q4 reaching this duration. Among no-biopsy procedures, only 3.8% reached 7 min, compared with 7.4% of procedures with at least one biopsy. At the physician level, only 4 of 213 physicians (1.9%) had a personal median procedural time of at least 7 min. When physicians were stratified into quintiles by their median procedural time, the proportion meeting the benchmark increased progressively from 1.2% in Q1 (fastest) to 15.5% in Q5 (slowest; Table 2 ).

### AI-Assisted Landmark Documentation

Among procedures performed with the AI timing platform active but without AI landmark suggestions ( *n* = 31,425), 27.5% had a zero-landmark count, and the median landmark count was 1 (IQR: 0–3). When AI landmark suggestions were additionally available ( *n* = 46,911), there were 15.8% zero-landmark procedures, a 42.6% reduction ( *P* < 0.001). Median landmark count was 3 (IQR: 1–4) versus 1 (IQR: 0–3) in the no-suggestion group ( *P* < 0.001). Mean landmarks per procedure: 1.97 vs. 2.65, a 34.7% difference ( *P* < 0.001; Fig. 2 ). Greater landmark documentation rate in the suggestion group was consistent across all procedural-time quintiles.

**Fig. 2:**
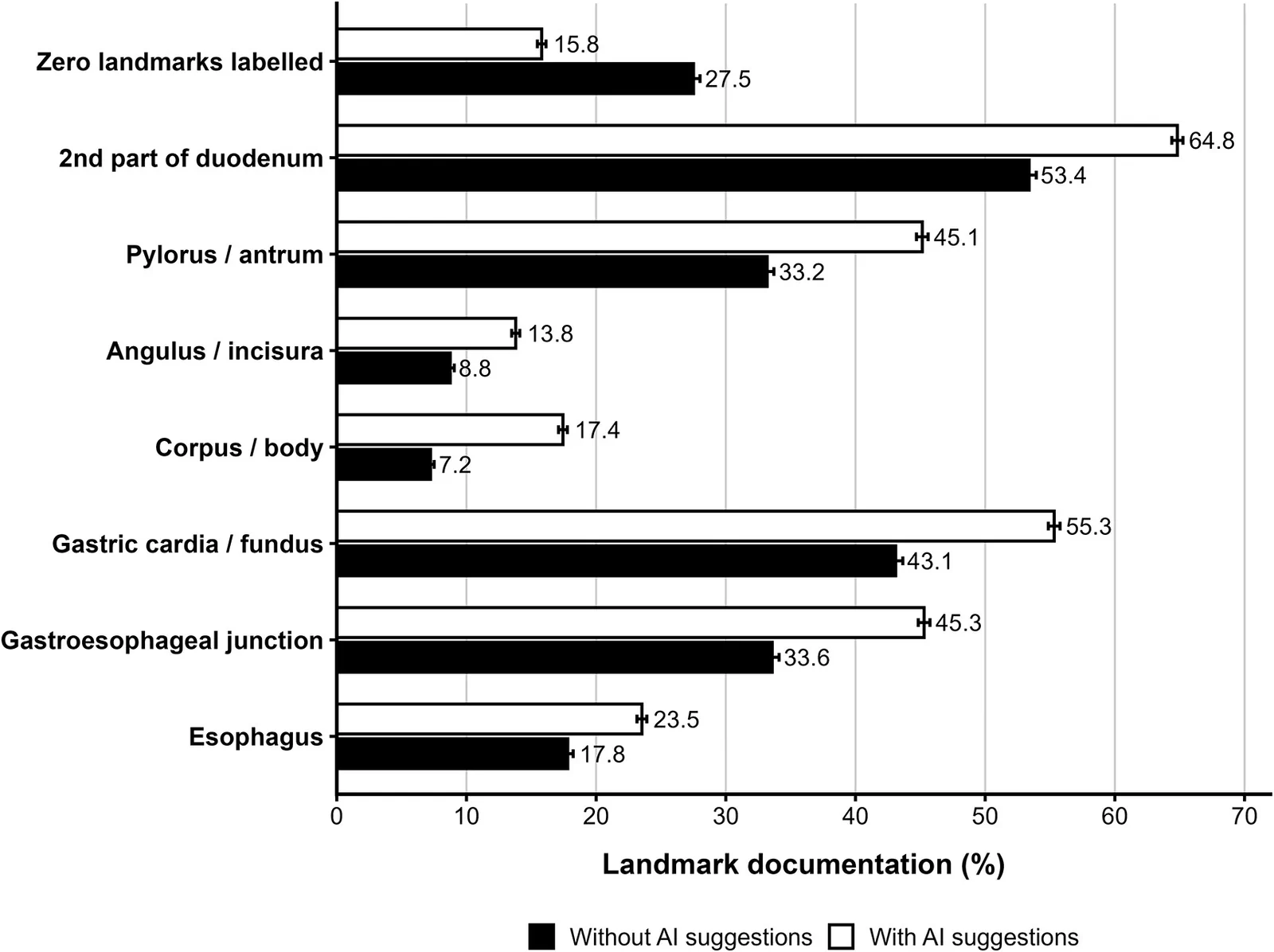
AI-assisted landmark documentation. Bar chart comparing documentation rates for each of the seven predefined anatomical landmarks in procedures without AI landmark suggestions ( *n* = 44,571) versus procedures with AI landmark suggestions ( *n* = 33,765). Error bars represent 95% confidence intervals. All between-group differences are statistically significant ( *P* < 0.001 by chi-square test). The overall proportion of procedures with zero documented landmarks decreased from 27.5% (without suggestions) to 15.8% (with suggestions). AI, artificial intelligence; pp, percentage points.

When landmark documentation rate was assessed individually by anatomical site, AI suggestions were associated with significantly higher documentation rates across all seven predefined landmarks ( *P* < 0.001 for each; Table 3 ). The largest absolute improvements were observed for the gastric cardia or fundus (+12.2 pp; 43.1 vs. 55.3%), pylorus or antrum (+12.0 pp; 33.2 vs. 45.2%), gastroesophageal junction (+11.7 pp; 33.6 vs. 45.3%), second part of the duodenum (+11.4 pp; 53.4 vs. 64.8%), and corpus or body (+10.2 pp; 7.2 vs. 17.4%). The second part of the duodenum was the most consistently documented landmark overall (60.2%), while the angulus or incisura (11.8%) and corpus or body (13.3%) had the lowest overall documentation rates, identifying these as anatomical sites most likely to benefit from systematic AI-assisted prompting.

**Table 3:** Individual landmark documentation rates by AI suggestion status.

Landmark	Overall (%)	Without AI suggestions ( *n* = 31,425)	With AI suggestions ( *n* = 46,911)	Absolute difference (pp)
Esophagus	21.2	17.8	23.5	+5.7*
Gastroesophageal junction	40.6	33.6	45.3	+11.7*
Gastric cardia/fundus	50.4	43.1	55.3	+12.2*
Corpus/body	13.3	7.2	17.4	+10.2*
Angulus/incisura	11.8	8.7	13.8	+5.1*
Pylorus/antrum	40.4	33.2	45.2	+12.0*
Second part of duodenum	60.2	53.4	64.8	+11.4*
Zero landmark procedures	20.5	27.5	15.8	−11.7*

* *P* < 0.001 by chi-square test. pp, percentage points.

### Endoscopic Findings and Procedural Time

Among the 69,754 procedures with available endoscopic diagnosis data, the most frequent findings were gastritis or gastropathy (38.1%), normal examination (27.0%), hiatal or diaphragmatic hernia (25.5%), esophagitis of any type (16.9%), and gastric, duodenal or esophageal polyps (8.7%). Categories are not mutually exclusive, as more than one diagnosis could be recorded for a single procedure. At the physician level, endoscopists in the slowest quintile of median procedural time recorded fewer normal examinations than those in the fastest quintile: 19.8 versus 31.7% ( *P* = 0.002; Spearman rho +0.18). Because total procedural duration includes time spent evaluating and sampling abnormalities, this comparison cannot by itself distinguish thorough inspection from lesion-related activity.

In a subanalysis using biopsy-free procedural time, the association observed in the main analysis was reproduced and was marginally stronger. Physicians in the slowest quintile of biopsy-free procedural time recorded a normal EGD in 20.1% of their procedures, compared with 34.5% in the fastest quintile (Spearman rho −0.22, *P* = 0.009; Jonckheere–Terpstra *P* = 0.009), against 19.8 and 31.7% in the main analysis. Intermediate quintiles were not ordered.

In sensitivity analyses, raising the qualifying threshold to 50 biopsy-free procedures (129 physicians) strengthened the association (17.0 vs. 35.4%; rho −0.29, *P* = 0.002), whereas lowering it to 20 procedures (174 physicians) attenuated it (19.4 vs. 33.1%; rho −0.19, *P* = 0.014).

### Age-Related Patterns and Variation within Age Groups

To determine whether physician-level variation reflects genuine differences in examination behavior rather than case mix, we examined two plausible confounders: patient age distribution and biopsy acquisition practice.

Procedural time varied significantly across age ranges (Kruskal–Wallis *H* = 1696.15; *P* < 0.0001). Pediatric patients aged 0–17 years ( *n* = 2,687) had the longest median procedural time at 4.25 min (IQR: 3.45–5.32). Young adults aged 18–29 years had the shortest at 3.00 min (IQR: 2.05–4.27). In adults, procedural time increased gradually with age from 3.00 min (30–39 years) to 3.50 min (70–79 years; Fig. 3A ).

**Fig. 3:**
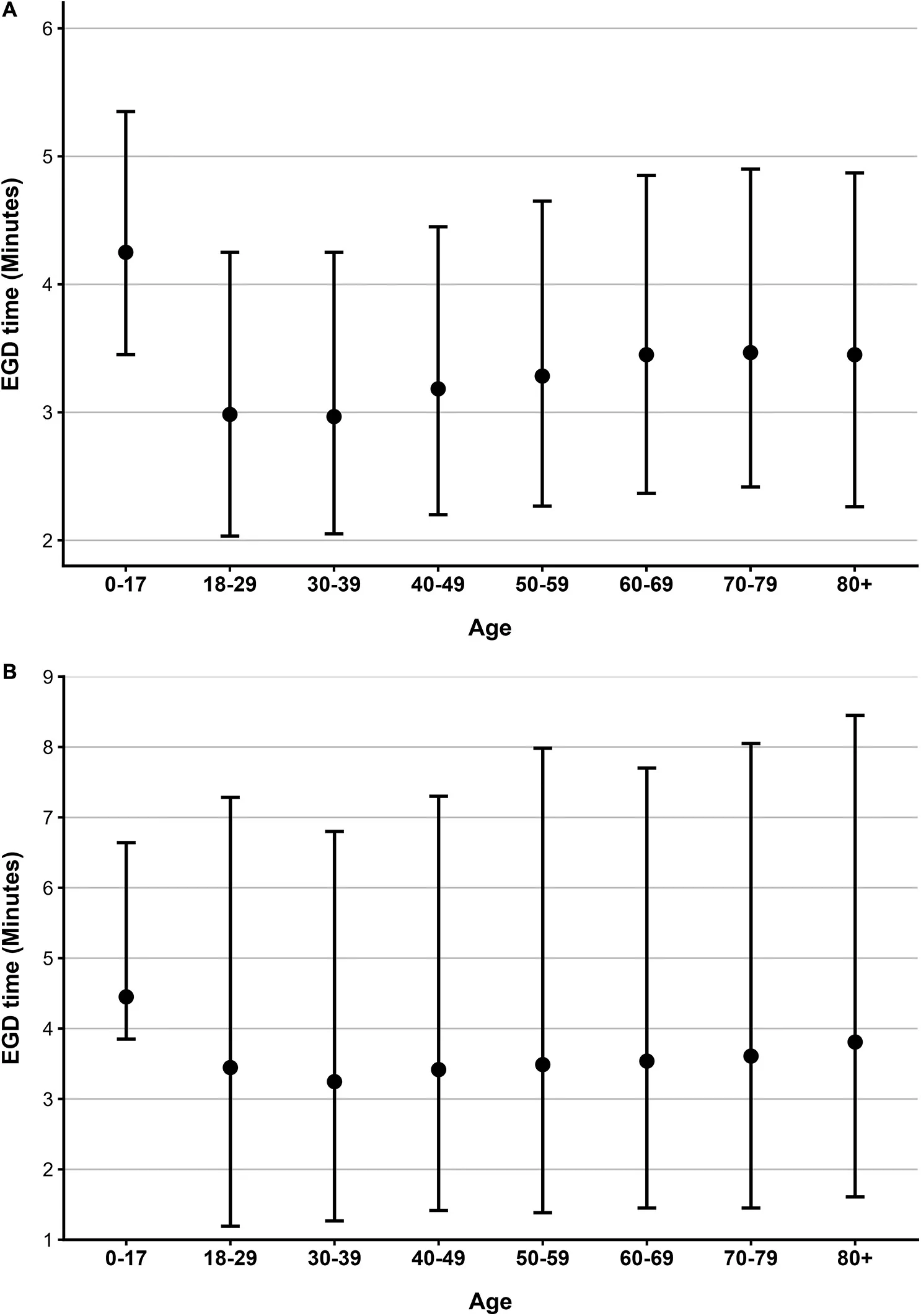
Procedural time variation across patient age groups and physician-level variation within age groups. ( **A** ) Median EGD procedural time by patient age category across the full cohort. Pediatric patients (0–17 years) had the longest median procedural time (4.25 min); young adults (18–29 years) had the shortest (3.00 min). ( **B** ) Physician-level variation in median procedural time within each age group. Each point represents a physician’s median procedural time for patients in that age category. Median procedural times varied 5.2- to 6.1-fold among physicians within the same age group. EGD, esophagogastroduodenoscopy; IQR, interquartile range.

Substantial physician-level variation persisted within each age group ( Fig. 3B ). Median procedural times varied 5.2- to 6.1-fold among physicians within the same age group. After direct age standardization, patient age explained only 6.5% of between-physician variance.

### Biopsy Acquisition and Biopsy-Practice Variation

Across all EGD procedures, 36.7% had no documented biopsies, 30.3% had 1 biopsy, 21.8% had 2 biopsies, and 11.2% had 3–5 biopsies (median 1, IQR: 0–2). Procedural time increased with biopsy count (Spearman *r* = 0.433; *P* < 0.0001), with median times of 2.60 min for no biopsy, 3.50 min for 1–2 biopsies, 5.05 min for 3–5 biopsies, and 7.53 min for more than 5 biopsies. Together, patient age and biopsy practice explained less than 20% of between-physician variation, indicating that the large majority of physician-level variation reflects genuine differences in examination behavior.

### Within-Physician Procedural-Time Stability

Within-physician analyses showed two distinct patterns ( Fig. 4 ). Across patient age groups, physicians maintained stable median procedural times (median CV: 9.4%; median fold difference: 1.28). In contrast, case-to-case variation was substantially greater (median CV: 44.3%; IQR: 36.5–52.9), with only 5.4% of physicians showing a CV below 30% ( Fig. 4B ).

**Fig. 4:**
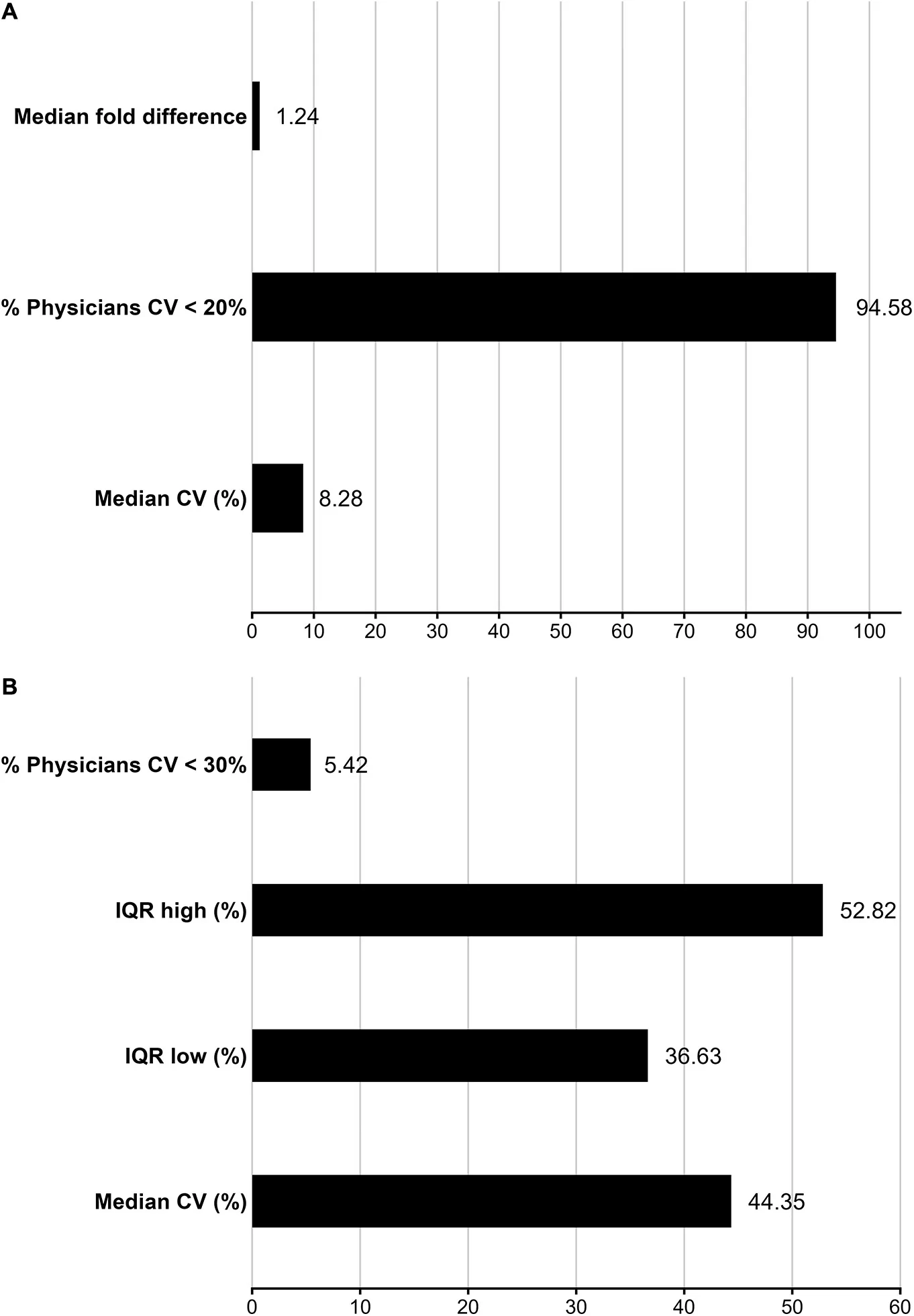
Within-physician procedural-time stability. ( **A** ) Coefficient of variation (CV) of each physician’s median procedural time across patient age groups, reflecting inter-age-group stability. Median CV was 9.4%, with 95.9% of physicians showing less than 20% variation across age groups. ( **B** ) CV of individual procedure times within each physician, reflecting case-to-case variability. Median CV was 44.3% (IQR: 36.5–52.9), substantially greater than inter-age-group variation. Only 5.4% of physicians had a case-to-case CV below 30%. CV, coefficient of variation.

## Discussion

This multicenter study, which encompassed 78,336 EGDs, explored the use of a video-based AI platform to automate procedural time measurement and enhance landmark documentation through real-time AI recommendations. The principal findings highlighted several important aspects: the AI-derived timestamps demonstrated a high level of validation accuracy; there was notable variability in procedural times among physicians, persisting despite case-mix adjustments; a compelling association was found between AI-suggested landmarks and the completeness of photodocumentation across all seven anatomical sites; and benchmarking against the ESGE’s 7-min threshold revealed that only 6.1% of procedures met this target. An exploratory analysis found longer procedural times associated with fewer normal examinations, an association that persisted when physicians were restratified using biopsy-free procedural time, although its direction cannot be established from these data. Taken together, these findings support the use of video-based AI as both an intraprocedural aid and a practical infrastructure for routine quality measurement. 9 10 11 12 13 14 15

The procedural duration findings are clinically relevant because upper endoscopy quality has traditionally been difficult to audit at scale. Several studies have shown that longer examination time is associated with improved detection of upper gastrointestinal neoplasia and focal lesions. 5 6 7 8 Recent guidelines recommend specific procedural duration times for EGD. 1 2 3 4 Automated detection of scope insertion and withdrawal is not, in itself, a technically demanding computer vision task, and we make no claim of algorithmic novelty; the underlying algorithm has been described previously. 16 The relevance of the present findings is operational. Procedural duration is a guideline-endorsed performance measure 2 3 4 that is nonetheless rarely captured in routine practice, being either unrecorded or recorded manually with recognized inaccuracy and with an observation effect once endoscopists are aware that timing is being audited. The 98.5% concordance with blinded expert review confirms that duration can be obtained passively and reliably for every consecutive procedure at scale, converting a recommended indicator into an auditable one suitable for individual feedback and institutional monitoring. 9 10 13 14

The observed median procedural time of 3.30 min, together with only 6.1% of procedures reaching 7 min, documents a substantial gap between routine community practice and the ESGE quality framework. Only 4 of 213 physicians (1.9%) had a personal median at or above the benchmark. This finding should be interpreted cautiously because the ESGE 7-min recommendation was established primarily in academic settings, and its applicability to routine community practice, where procedural volumes, patient mix, and workflow conditions differ substantially, remains to be validated. 3 4 5 6 7 8 National quality frameworks differ in the standards they set for diagnostic upper endoscopy, 17 and consensus standards for neoplasia-directed examination in high-incidence settings are more demanding still. 18 25 The progressive increase in benchmark attainment from 1.2% in the fastest physician quintile to 15.5% in the slowest demonstrates that AI-derived procedural time meaningfully captures the distribution of examination practice and identifies physicians most likely to benefit from quality improvement interventions.

A key methodological question is whether physician-level variation reflects genuine differences in examination behavior or differences in case mix. Patient age explained only 6.5% of the between-physician variance, and patient age and biopsy practice together explained less than 20% of the variation. We also examined the relationship between procedural duration and documented endoscopic diagnoses. Physicians with longer median procedural times recorded fewer normal examinations, and at the procedure level, examinations with a documented finding were longer than normal examinations (median 3.48 vs. 2.78 min; *P* < 0.001). Neither observation establishes the direction of the relationship, because evaluating, photographing, and sampling an abnormality itself consumes time, and the endoscopist who samples more will therefore appear slower irrespective of inspection thoroughness.

To address this, we restratified physicians using biopsy-free procedural time, measured only on procedures in which no tissue was taken, and then examined diagnostic rates across all of their procedures ( Table 4 ). The association was reproduced and was marginally stronger than in the main analysis, and it strengthened further when the exposure was restricted to physicians with more qualifying procedures, a pattern consistent with attenuation by measurement error rather than with an artifact of sampling time. This indicates that the relationship between physician procedural time and documented findings is not explained by biopsy-related activity. Important limits remain. Diagnoses were endoscopist-assigned report codes without histopathologic confirmation, and abnormalities that are characterized or photographed without sampling still consume time, so the dependency is reduced rather than eliminated. Intermediate quintiles were not ordered, which argues against interpreting the relationship as a simple dose–response. These analyses are exploratory and hypothesis-generating.

**Table 4:** Normal-EGD rate by quintile of biopsy-free procedural time.

Quintile	Physicians	Procedures	Biopsy-free time, median (range), min	Normal EGD (%)	Abnormal (%)
Q1 (Fastest)	33	16,633	1.68 (1.13-2.05)	34.5	65.5
Q2	28	11,273	2.20 (2.06-2.44)	26.4	73.6
Q3	30	10,479	2.61 (2.45-2.85)	27.3	72.7
Q4	31	10,749	3.19 (2.88-3.42)	31.0	69.0
Q5 (Slowest)	29	8,974	4.10 (3.45-6.63)	20.1	79.9

Physicians with at least 30 procedures in which no biopsy was taken ( *n* = 151) were stratified into quintiles of their median biopsy-free procedural time. Diagnostic rates were calculated across all procedures performed by those physicians for which diagnosis data were available ( *n* = 58,108), including procedures in which a biopsy was taken. Spearman rho -0.22, *P* = 0.009; Jonckheere–Terpstra test for trend *P* = 0.009.

The photodocumentation findings are a central contribution of this study. Increasing recognition of systematic landmark imaging as a core component of high-quality upper endoscopy is reflected in recent guidelines and position statements. 2 3 4 21 Prior AI studies in this area have focused on anatomical site classification, photodocumentation quality scoring, and automated image reporting. 11 22 23 24 The present study provides large-scale pragmatic evidence showing that AI-generated landmark suggestions were associated with a 42.6% reduction in zero-landmark procedures and a higher median landmark count during routine practice. The individual landmark analysis revealed significant improvements across all seven anatomical sites, with the largest gains at the gastric cardia/fundus, pylorus/antrum, and gastroesophageal junction: anatomically demanding sites where documentation is most likely to be omitted during a hurried examination. The angulus/incisura and corpus/body had the lowest baseline documentation rates (8.7 and 7.2%, respectively, without AI), rising significantly with AI suggestions (13.8 and 17.4%). These data provide granular evidence of where AI-assisted prompting adds the greatest clinical value in upper endoscopy photodocumentation practice. The consistency of this effect across all procedural-time quintiles supports a genuine workflow-level effect on documentation behavior, rather than a simple correlation with longer examination time. 9 10 11 24

Broadly, the current findings are at the intersection of clinical quality literature showing that examination time, photodocumentation, and operator behavior are meaningfully associated with EGD quality 5 6 7 8 19 20 and AI literature showing that real-time AI-based systems can reduce blind spots, improve procedural completeness, and automate reporting and documentation. 9 10 11 12 13 14 15 The main contribution is making this connection at an operational scale across routine multicenter practice, with individual landmark-level evidence that has not previously been reported in this setting.

Several limitations should be acknowledged. First, this was a retrospective observational study. Second, procedural time included biopsy-related and other intraprocedural activity rather than pure mucosal inspection time. Third, these findings describe a single Israeli service, and their transferability requires careful framing. Assuta Medical Centers function as the procedural arm of one of Israel’s national health funds, and the great majority of the examinations analyzed here were performed on patients insured under the national health system, evaluated by community gastroenterologists, and referred for endoscopy under public reimbursement, with only a minority privately funded. The cohort therefore reflects routine, referral-based, publicly funded practice for a large national population rather than a self-pay group, and the case mix is weighted toward symptomatic outpatient indications rather than population-based gastric cancer screening. These findings should not be extrapolated to high gastric cancer incidence settings such as Japan or Korea, where dedicated screening protocols and substantially longer inspection times are standard, nor to academic teaching units. What these data do establish is that when procedural duration is captured automatically and without observation effect across every consecutive procedure in a large national provider, routine practice may fall well below current recommendations. Payer status was not recorded at the procedure level, and we could not examine whether procedural time or documentation completeness differed between publicly and privately funded examinations. In addition, the cohort is drawn predominantly from the membership of a single national health fund, and referral criteria, reimbursement arrangements, and scheduling practices specific to that system may have influenced procedural volumes and duration. Fourth, unmeasured factors such as the indication for EGD, sedation practices, and local workflow may have contributed to the observed variation. Fifth, diagnostic data were available for 69,754 of 78,336 procedures owing to incomplete linkage between the AI platform and the clinical reporting system. Diagnoses were endoscopist-assigned report codes without histopathologic confirmation and are subject to interobserver variation in diagnostic threshold and terminology; neoplasia detection rates cannot be inferred from these data. Therapeutic interventions were not available as a discrete field in the platform export, and the platform measures total in-body time without separating inspection from intervention, so time spent evaluating or treating a detected lesion could not be isolated. Biopsy count was the only available proxy for procedural work. The biopsy-free subanalysis reduces but does not eliminate this dependency, since abnormalities characterized or photographed without sampling still consume time, and it retained 151 of 213 physicians. All analyses of documented endoscopic diagnoses should therefore be regarded as exploratory and hypothesis-generating. Sixth, our photodocumentation analysis assessed completeness only. Whether AI-prompted images meet accepted standards of diagnostic adequacy, for example those set out in the World Endoscopy Organization position statement, 21 was not evaluated. We did not assess image resolution, adequacy of insufflation or mucosal cleanliness, field of view, or image clarity. A higher landmark count therefore demonstrates more complete documentation rather than better documentation, and it remains possible that prompted images are captured more perfunctorily than images acquired on the endoscopist’s own initiative. A blinded assessment of image adequacy is a necessary next step before AI-assisted photodocumentation can be regarded as a validated quality intervention. Seventh, we were unable to characterize endoscopist experience or unit type. The platform export contained a de-identified physician identifier without years since certification, subspecialty focus, or prior procedural experience, and institutional approval did not extend to linkage with personnel records. Procedural volume was not used as a proxy because it is arithmetically dependent on procedural speed, the exposure under study. All units in the network are nonacademic service units, so comparison between day centers, hospital units, and university teaching units was not possible, and our findings should not be assumed to apply to training environments in which supervised procedures are systematically longer. Variation between endoscopists may therefore reflect experience, subspecialty focus, or case complexity in addition to inspection thoroughness.

Finally, because the landmark documentation analysis was based on phased rollout rather than randomized allocation, the observed improvement should be interpreted as a strong real-world association rather than definitive proof of causality.

In conclusion, video-based artificial intelligence has demonstrated effectiveness in accurately measuring EGD procedure durations and enhancing landmark documentation rate through AI-generated recommendations. The observed median procedural time of 3.3 min indicates a significant gap regarding quality standards. Automated capture of procedural duration and landmark documentation offers a practical basis for routine quality auditing in upper endoscopy.

## Data Availability

Data is not available due to confidentiality.

## Declaration of GenAI Use

The authors used Claude Opus 5 for editing.
